# The Occurrence of *Borrelia burgdorferi* sensu lato in *Ixodes ricinus* Ticks Collected from Nature-Educational and Tourist Trails in the Poprad Landscape Park

**DOI:** 10.3390/pathogens14020117

**Published:** 2025-01-26

**Authors:** Sylwia Koczanowicz, Magdalena Nowak-Chmura, Anna Kocoń, Grzegorz Rączka, Marek Asman

**Affiliations:** 1 Department of Zoology, Institute of Biology and Earth Sciences, University of the National Education Commission, Podchorążych 2, 30-084 Cracow, Poland; 2 Department of Medical and Molecular Biology, Faculty of Medical Sciences in Zabrze, Medical University of Silesia, Jordana 19, 41-808 Zabrze, Poland; 3 Department of Forest Management Planning, Poznań University of Life Sciences, Wojska Polskiego 28, 60-637 Poznań, Poland

**Keywords:** ticks, *Ixodes ricinus*, *Borrelia burgdorferi* s.l., Poprad Landscape Park

## Abstract

Throughout Europe, including Poland, *Ixodes ricinus* ticks are the main vector of numerous pathogenic agents that pose a serious threat to public health. Southern Poland attracts many tourists with its scenic landscapes and abundant recreational opportunities. These areas are ideal habitats for wild fauna, which serve as the main reservoirs and hosts for these pathogens and ticks. The large population and biodiversity of these hosts facilitate the proliferation of ticks. The aim of this study was to determine the potential exposure of humans to ticks and tick-borne pathogens such as *Borrelia burgdorferi* sensu lato, *Anaplasma phagocytophilum*, and *Babesia* spp., along the nature-educational and tourist trails of the Poprad Landscape Park. From 2020 to 2021, ticks were collected using the flagging method on three tourist trails and nature-educational paths within the Poprad Landscape Park. DNA was isolated from 213 *I. ricinus* ticks using the ammonia method. To detect pathogens in ticks, PCR and nested PCR methods were used. To detect *B. burgdorferi* s.l. and *A. phagocytophilum*, two pairs of primers specific to the *flaB* gene fragment and 16S rRNA gene fragment were used, respectively. For *Babesia* spp. detection, primers specific to the 18S rRNA gene were used. The amplification products were separated electrophoretically and visualized under ultraviolet light. In total, among the 213 examined ticks, *B. burgdorferi* s.l. was detected in 31% of the samples. Neither *A. phagocytophilum* nor *Babesia* spp. were detected in the studied material. These results indicate a potentially high risk of ticks and tick-borne *B. burgdorferi* s.l. infections for residents and tourists in the recreational areas of the Poprad Landscape Park.

## 1. Introduction

Poprad Landscape Park is located in southern Poland, within the Lesser Poland Voivodeship. Situated in the Western Carpathians, specifically the Beskid Sądecki range, it encompasses the Poprad river valley. It is the largest landscape park in the voivodeship and one of the largest in Poland, covering over 53,000 hectares and spanning both Poland and Slovakia. Thanks to its rich biodiversity and unique landscapes, the park attracts numerous visitors, including local residents as well as domestic and international tourists. Frequent visits by tourists increase their risk of exposure to ticks and tick-borne diseases. Most of the park’s area is covered by mixed forests, and the presence of various species of wild animals serving as hosts for ticks makes it an ideal habitat for the development of *Ixodes ricinus* ticks [1,2].

*Ixodes ricinus* is a tick species of significant medical and veterinary importance, widely distributed in Poland, including its southern regions [3]. The primary reservoirs of tick-borne pathogens are small mammals, such as mice and voles, various bird species, and larger animals, including deer. These hosts sustain pathogens in the environment and play a crucial role in the tick’s life cycle and disease transmission. Tick-borne diseases are primarily transmitted through the bite of an infected tick. During feeding, ticks inject saliva containing pathogens, including bacteria, viruses, or parasites, into the host’s bloodstream. Tick saliva contains anticoagulants and immunosuppressants, which facilitate pathogen transmission by evading the host’s immune response [4].

Both in Europe and specifically in Poland, *I. ricinus* serves as the primary vector of *Borrelia burgdorferi* sensu lato, the etiological agent of Lyme borreliosis, which is the most common tick-borne disease affecting humans in the Northern Hemisphere [5]. The increase in Lyme disease cases observed in recent years may suggest the presence of favorable conditions for spirochetes and their vectors [6]. Moreover, in 2023, a record number of Lyme disease cases was recorded in Poland, totaling 25,285 cases, with an incidence rate of 67.1 per 100,000 population, and 4.6% of the cases required hospitalization [7]. This disease poses a significant health risk due to its widespread prevalence and potential for severe complications. It can manifest with diverse symptoms, including erythema migrans, fever, muscle and joint pain, and, in later stages, neurological or cardiac complications. This is a multisystem disease that is difficult to diagnose and treat, often requiring a multidisciplinary approach [8]. Another significant tick-borne disease, anaplasmosis, is caused by *Anaplasma phagocytophilum*. This pathogen targets white blood cells, disrupting the immune response and leading to symptoms such as fever, muscle pain, and fatigue. In severe cases, particularly in immunocompromised individuals, anaplasmosis can result in organ damage [9]. Babesiosis, another well-known tick-borne disease, is caused by protozoa of the genus *Babesia*. These parasites infect and destroy red blood cells, leading to symptoms such as fever, chills, anemia, and fatigue, which closely resemble malaria. Severe cases are more likely to occur in individuals with weakened immune systems or those lacking a spleen, potentially progressing to organ failure [10].

Although research has been conducted in southern Poland for years, sufficient information on the occurrence of ticks in many tourist areas, including the Poprad Landscape Park, is still lacking. It is crucial to conduct research on the presence of ticks in tourist areas and analyze the pathogens of tick-borne diseases that these parasites carry, enabling the identification of potential public health threats and contributing to the development of effective prevention and treatment methods for tick-borne diseases [11,12,13]. Consequently, the aim of this study was to assess the potential risk of human exposure to ticks and tick-borne pathogens, including *B. burgdorferi* s.l., *A. phagocytophilum*, and *Babesia* spp., along the selected nature-educational and tourist trails of the Poprad Landscape Park.

## 2. Materials and Methods

### 2.1. Sampling Area

**Figure 1:**
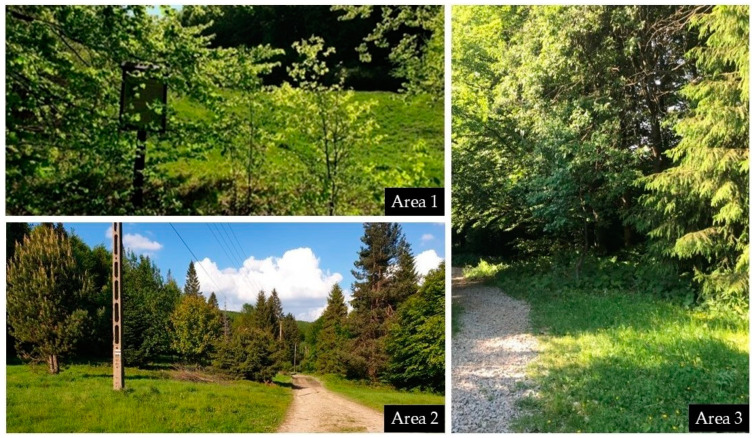
Examples of tick collection sites within the Poprad Landscape Park.

The Poprad Landscape Park is predominantly covered with forests and holds significant ecological importance, encompassing numerous protected areas, including 13 nature reserves. In this study, the area is categorized into two distinct climatic altitudinal vegetation zones: the foothill zone and the lower forest zone. In the foothill zone, cultivated fields, meadows, and pastures dominate, interspersed with larger patches of forests and groves. This zone is characterized by the presence of xerothermic plants as well as alder carrs and reed beds in river valleys. In contrast, the lower forest zone primarily consists of beech forests with mixtures of fir, sycamore, mountain elm, and spruce. In the spring, this zone is rich with a variety of plants including snowdrops, glandular liverleaf, and wild garlic. The park’s forests are home to a wide variety of wildlife, including lynxes, wolves, deer, wildcats, and bears, as well as providing a habitat for mountain bird species such as the golden eagle, Ural owl, ring ouzel, and rock thrush. Additionally, the valleys of the Poprad and Dunajec rivers serve as habitats for otters and beavers, further reflecting the park’s rich biodiversity and environmental significance. Area 1—the “Rogasiowy Szlak” nature path in Rytro is 12.4 km long, with an elevation range from 443 to 1002 m above sea level. The sampling in this area was conducted in 2020. It features 14 educational boards along the route. This area is predominantly covered by beech and fir-pine trees. Area 2—“Barani Szlak” in Rytro is 4.2 km long, with an elevation range from 569 to 827 m a.s.l. The sampling in this area was carried out in 2021. This trail passes through mixed forests, meadows, and fields. The trail begins at the castle and follows the path to the mountain shelter. Area 3—“Na Stoku Jaworzyny Krynickiej” nature-educational path is 4.5 km long, with an elevation range from 720 to 1100 m a.s.l. The sampling in this area was conducted in 2021. It includes 14 thematic stops that describe the area’s flora, fauna, and geology. The path leads through diverse landscapes, including beech-fir forests and meadows (Figure 1).

### 2.2. Field Sampling

**Figure 2:**
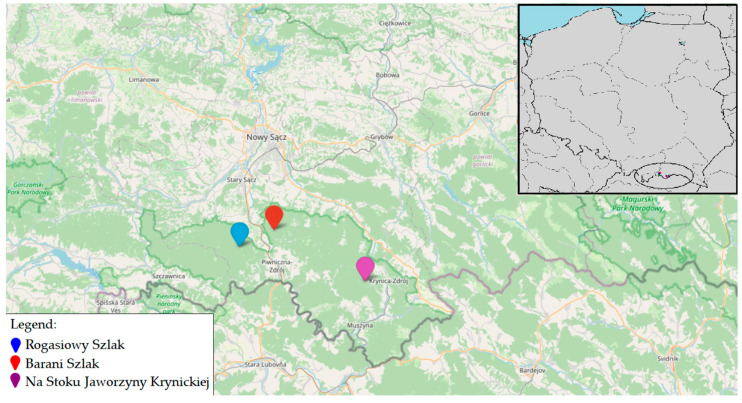
Locations of studied trails and nature-educational paths in the Poprad Landscape Park.

The collection of ticks from each research area (Figure 2) was conducted during spring, specifically in June, of 2020 and 2021. In 2020, 72 ticks were gathered from the “Rogasiowy Szlak” trail (6 males, 7 females, and 59 nymphs); in 2021, 110 ticks were collected along the “Barani Szlak” trail (50 males, 41 females, and 19 nymphs), and also in 2021, 31 ticks were collected from the nature-educational path “Na Stoku Jaworzyny Krynickiej” (5 males, 11 females, and 15 nymphs). Along each research area, 4 sampling points were designated, with the first point marking the beginning of the trail and the last marking its end. The collection on each trail lasted 40 min, with 10 min at each sampling point. It was performed using the flagging method by a single individual.

### 2.3. Laboratory Analysis

#### 2.3.1. Ticks

The collected ticks were placed in tubes filled with 70% ethyl alcohol. Subsequently, the ticks were identified by species and developmental stage under a stereoscopic microscope using the tick identification key by Siuda [3].

#### 2.3.2. PCR

The DNA was isolated from the single ticks using the ammonia method [14]. Its concentration was measured spectrophotometrically in a NanoPhotometer PEARL (Implen, Munich, Germany) at 260/280 nm wavelength. Next, samples were frozen at −20 °C and stored for further molecular analysis. To detect *B. burgdorferi* s.l. in ticks, two pairs of primers specific to the *flaB* gene fragment were used (Table 1) [15]. For the amplification, a DNA quantity of 200 ng and Maximo DFS Plus Taq DNA Polymerase (GeneOn, Ludwigshafen, Germany) were used. In turn, for the re-amplification, 1 µL of PCR product was added. *Anaplasma phagocytophilum* was detected in the studied material using primers specific to the 16S rRNA gene fragment (Table 1) [16]. For the amplification of 200 ng of DNA, Taq DNA Polymerase (EURx, Gdańsk, Poland) was used. In turn, for the re-amplification, 1 µL of PCR product was added. Protozoan *Babesia* spp. were detected in ticks using a pair of primers specific to the 18S rRNA gene fragment (Table 1) [17]. For the amplification, 200 ng of DNA and Taq DNA Polymerase (EURx, Gdańsk, Poland) were used. The amplification products were separated electrophoretically in 2% ethidium bromide-stained agarose gels and visualized under ultraviolet light. The presence of PCR products with sizes of 774 base pairs [bp] and 605 bp for *B. burgdorferi* s.l., 932 bp and 546 bp for *A. phagocytophilum*, and 620 bp for *Babesia* spp. were considered positive (Table 1). The positive controls of studied pathogens were sourced from the collection of the Department of Medical and Molecular Biology, Faculty of Medical Sciences in Zabrze at the Medical University of Silesia, while the negative was DNA/RNA free water.

### 2.4. Statistics

To verify the significance of differences in the number of infected and uninfected ticks, χ^2^ tests (Chi-square tests) were used. All the calculations were conducted using TIBCO Software Inc. (2017, Palo Alto, CA, USA). Statistica (data analysis software system), version 13.

## 3. Results

Altogether, 213 ticks (61 males, 59 females, and 93 nymphs) were collected from three collection sites in spring of 2020 and 2021. In total, *B. burgdorferi* s.l. was detected in 31.0% of studied *I. ricinus* ticks (n = 66) and the number of infected ticks was lower than the number of uninfected ones (n = 147, 69.0%). The presence of this spirochete was shown in two out of three studied tourist trails. The highest prevalence, 43.1%, was observed in *I. ricinus* collected in Area 1. In turn, in ticks collected from Area 2, the prevalence of this bacterium was lower, at 31.8%, while there were no ticks with *B. burgdorferi* s.l. detected in Area 3. The presence of *A. phagocytophilum* and *Babesia* spp. was not detected in the studied material (Table 2). Results of the χ^2^ test did not show statistically significant differences (*p* < 0.05) between the proportions of the number of infected and uninfected ticks representing Area 1 and Area 2, χ^2^ (1, N = 182) = 2.377; *p*-value = 0.123. Area 3 was excluded from the test because the number of infected ticks was zero.

Generally, *B. burgdorferi* s.l. was found in all developmental stages of *I. ricinus* that were studied (20 males, 19 females, and 27 nymphs). The prevalence was higher in adult ticks than in nymphs, at 32.5% and 29.0%, respectively. The overall frequency of *B. burgdorferi* s.l. in males and females of *I. ricinus* was only slightly different, amounting to 32.8% and 32.2%, respectively (Table 2). The proportions of the number of infected and uninfected ticks in terms of the two developmental stages (mature ticks and nymphs) did not show statistically significant differences (*p* < 0.05), χ^2^ (1, N = 213) = 0.295; *p*-value = 0.587. Similarly, no statistically significant differences were observed between the sexes of mature individuals (males and females), χ^2^ (1, N = 213) = 0.005; *p*-value = 0.946.

The entire tick collection (N = 213) was also divided into two groups representing two different climatic altitudinal vegetation zones: the foothill zone, to 600 m a.s.l. (n = 32), and the lower forest zone, from 600 m a.s.l. (n = 181), in which the percentage of ticks infected with *B. burgdorferi* s.l. was 34.4% and 30.4%, respectively. Results of the χ^2^ test did not show statistically significant differences (*p* < 0.05) between the proportions of the number of infected and uninfected ticks in these zones, χ^2^ (1, N = 213) = 0.202; *p*-value = 0.653.

## 4. Discussion

*Ixodes ricinus* ticks are more commonly found at lower elevations, where populations of their primary hosts are more abundant. In our study, ticks were found in both the foothill and lower forest zones. The highest potential risk of human tick infestation was observed along the Barani Szlak in Rytro. This trail is characterized by the smallest elevation difference between its starting and ending points. Additionally, the surveyed area ends at a relatively low altitude of 827 m above sea level, whereas other trails reach heights exceeding 1000 m. A slightly lower tick count was observed along the educational Rogasiowy Szlak in Rytro, which is notable for its relatively low altitude, especially at its starting point. Conversely, the lowest number of ticks was found on the highest trail Na Stoku Jaworzyny Krynickiej. *Ixodes ricinus* ticks have previously been recorded at similar or higher elevations. In Croatia, they were observed at an altitude of 1000 m above sea level [18], in the Czech Republic at 1250 m [19], and in Italy up to 1824 m [20]. Further detailed research in subsequent years will be necessary to understand the relationships between altitudes, zones, and tick prevalence.

*Borrelia burgdorferi* sensu lato is the most frequently studied and detected pathogen in southern Poland. The prevalence of this pathogen in southern Poland varies significantly, ranging from 0% to as high as 75% [21,22]. High prevalence values of this spirochete were also detected in the selected recreational regions of Beskid Żywiecki, reaching 62% [23]. In this study, these bacteria were detected in 31% of examined *I. ricinus* ticks, confirming their presence in all studied developmental stages of this ectoparasite. The prevalence of *B. burgdorferi* s.l. was found to be higher in adult ticks compared to nymphs, with respective infection rates of 32.5% and 29.0%. The proportions of infected and uninfected ticks across the two developmental stages—adult ticks and nymphs—did not show a statistically significant difference. Among adult *I. ricinus* ticks, the frequency of infection exhibited minimal variation between gender, with males and females showing infection rates of 32.8% and 32.2%, respectively. A similar overall prevalence (22–28.75%) for *B. burgdorferi* s.l. was demonstrated in a study conducted in the mountainous and foothill regions of southern Poland [24,25] and even recreational areas in northeastern and eastern Poland (20–25.2%) [26,27]. Additionally, previous studies conducted in other selected recreational areas of the Poprad Landscape Park in the years 2018–2019 showed the presence of *B. burgdorferi* s.l. in nearly three times fewer ticks—11.8%, with the bacteria confirmed in all studied developmental stages—males, females, and nymphs of *I. ricinus*. Moreover, differences in the prevalence of *B. burgdorferi* s.l. were observed between the studied areas, ranging from 4.9% to 27.5% [28]. In this study, the differences between studied areas were greater, ranging from 0–43.1%. The highest prevalence of *B. burgdorferi* s.l. (43.1%) was recorded in ticks collected from the “Rogasiowy Szlak,” an area covered with beech-fir forest. Furthermore, the study revealed that 100% of male ticks from this location carried the pathogen. A slightly lower prevalence (31.8%) of *B. burgdorferi* s.l. was detected along the “Barani Szlak”. *B. burgdorferi* s.l. spirochetes were not detected only on the “Na Stoku Jaworzyny Krynickiej” nature-educational path in Krynica-Zdrój. Additionally, the infection rates of *B. burgdorferi* s.l. were 34.4% and 30.4%, respectively, in these zones. No statistically significant differences were demonstrated between the proportions of infected and uninfected ticks. These differences in the prevalence of *B. burgdorferi* s.l., between the studied areas may be due to, among other factors, the high number and biodiversity of hosts in this area contributing to maintaining a higher infection level within the tick population [29]. Moreover, the study conducted by Tarageľová et al. [30] showed that the number of ticks infected with *B. burgdorferi* s.l. decreases with altitude. This fact may also explain the differences in tick infection with this spirochete on the studied nature-educational and tourist trails in the Poprad Landscape Park. A high percentage of ticks infected with *B. burgdorferi* s.l. indicates the need to conduct further, more detailed studies on the occurrence of genospecies of this bacterium in the studied areas. So far, nine genospecies of this bacterium have been detected both in *I. ricinus* and its hosts in Poland: *Borrelia afzelii, Borrelia bavariensis*, *Borrelia burgdorferi* sensu stricto, *Borrelia garinii*, *Borrelia lusitaniae*, *Borrelia spielmanii*, *Borrelia valaisiana*, *Borrelia bissettii* and *Borrelia turdi* [31,32]. In addition to these, *Borrelia miyamotoi* has also been detected in this tick species [33]. In Poland, the dominant genospecies is *B. afzelii*, the main reservoir of which are rodents [34,35]. This genospecies of spirochete is among those responsible for causing specific skin symptoms in humans. In addition to this genospecies, two other genospecies of this bacterium cause characteristic symptoms in humans: *B. burgdorferi* s.s. causes joint symptoms and *B. garinii* causes neurological symptoms [36]. Such in-depth analysis, including a larger number of ticks as well as their hosts, would be important to assess the actual threat of Lyme disease in these areas, allowing for the development of more effective prevention and intervention strategies. It would also help to adapt local health programs to combat this growing public health problem.

In the current research, *A. phagocytophilum* and *Babesia* spp. were not detected in *I. ricinus* ticks. The lack of detection of these pathogens due to the limited number of ticks studied does not mean that they are absent from the area, but rather that the research should be expanded and continued on a larger scale. In other mountainous areas, studies on ticks collected from vegetation have reported *Babesia microti* prevalence rates of 35% in the Żywiec Landscape Park and 58.32% in the Tarnowskie Góry District [23,37]. For *A. phagocytophilum*, the prevalence rates were 5% in the Żywiec Landscape Park and 32.7% in the Jeleśnia Municipality in the Żywiec Beskids [23,38]. Moreover, previous studies conducted in other selected recreational areas of the Poprad Landscape Park revealed a low overall prevalence of 0.3% for *A. phagocytophilum* and a higher prevalence of 7.4% for *B. microti* [28]. The results obtained in this study may confirm a low potential risk of human infection with *A. phagocytophilum* in the surveyed areas of the Poprad Landscape Park. *I. ricinus* ticks at higher altitudes encounter additional challenges, such as lower temperatures and shorter snow-free periods. These conditions shorten their active period, reducing the likelihood of locating a host and ultimately resulting in lower population densities [39]. Furthermore, limited access to reservoirs in the form of hosts may restrict the spread of pathogens carried by these ticks. Nonetheless, the occurrence of pathogen-infected ticks in recreational urban and suburban areas poses a direct threat to human and animal health [40,41].

## 5. Conclusions

This study confirmed the presence of *I. ricinus* ticks at various altitudes. Furthermore, the obtained results indicate a high risk of potential human exposure to tick-borne infections with *B. burgdorferi* s.l. The park’s environment provides favorable conditions for the presence of ticks. Both humans and animals are at risk of tick exposure and tick-borne diseases. Given that Lyme disease is becoming an increasingly common illness, it is essential to raise awareness among tourists and residents about the potential health risks and to promote the importance of adhering to preventive measures. These findings underscore the need for further studies and long-term monitoring of additional areas to understand the distribution of ticks and the mechanisms of pathogen transmission. The results may help to define educational and preventive measures aimed at people visiting these areas.

## Figures and Tables

**Table 1:** Oligonucleotide primers and PCR conditions used in the detection of *Borrelia burgdorferi* sensu lato [8], *Anaplasma phagocytophilum* [9], and *Babesia* spp. [10] in ticks collected from the selected tourist trails in the Poprad Landscape Park.

Primer	Sequence (5′–3′)	Gene Detected	Size of Amplification Product [bp]	PCR Conditions [°C/s]			N° of Cycles
				Denaturation	Annealing	Extension	
132f	TGGTATGGGAGTTTCTGG	*flaB*	774	94/30	50/45	72/60	40
905r	TCTGTCATTGTAGCATCTTT						
220f	CAGACAACAGAGGGAAAT		605	94/30	54/45	72/60	40
824r	TCAAGTCTATTTTGGAAAGCACC						
ge3a	CACATGCAAGTCGAACGGAT TATTC	16S rRNA	932	94/30	55/30	72/60	40
ge10r	TTCCGTTAAGAAGGATCTAATCTCC						
ge9f	AACGGATTATTCTTTATAG CTTGCT		546	94/30	55/30	72/60	30
ge2	GGCAGTATTAAAAGCAGCTCCAGG						
Babfor	GACTAGGGATTGGAGGTC	18S rRNA	620	94/60	53/45	72/90	35
Babrev	GAATAATTCACCGGATCACTC						

**Table 2:** The total number and percentage of *Ixodes ricinus* developmental stages infected with *Borrelia burgdorferi* sensu lato collected from the selected tourist trails in the Poprad Landscape Park.

Collection Site *	Developmental Stage	Number of Studied Ticks	Number and Percentage of Uninfected Ticks	Number and Percentage of Ticks Infected with *Borrelia burgdorferi* sensu lato
1	Male	6	0 (0.0%)	6 (100.0%)
	Female	7	6 (85.7%)	1 (14.3%)
	Nymph	59	35 (59.3%)	24 (40.7%)
	Total	72	41 (56.9%)	31 (43.1%)
2	Male	50	36 (72.0%)	14 (28.0%)
	Female	41	23 (56.1%)	18 (43.9%)
	Nymph	19	16 (84.2%)	3 (15.8%)
	Total	110	75 (68.2%)	35 (31.8%)
3	Male	5	5 (100.0%)	0 (0.0%)
	Female	11	11 (100.0%)	0 (0.0%)
	Nymph	15	15 (100.0%)	0 (0.0%)
	Total	31	31 (100.0%)	0 (0.0%)
1–3	Male	61	41 (67.2%)	20 (32.8%)
	Female	59	40 (67.8%)	19 (32.2%)
	Nymph	93	66 (71.0%)	27 (29.0%)
	Grand total	213	147 (69.0%)	66 (31.0%)

* Explanations: 1—Rogasiowy Szlak; 2—Barani Szlak; 3—Na Stoku Jaworzyny Krynickiej.

## Data Availability

The original contributions presented in the study are included in the article; further inquiries can be directed to the corresponding authors.
